# A comparative quantitative study of utilizing artificial intelligence on electronic health records in the USA and China during 2008–2017

**DOI:** 10.1186/s12911-018-0692-9

**Published:** 2018-12-07

**Authors:** Xieling Chen, Ziqing Liu, Li Wei, Jun Yan, Tianyong Hao, Ruoyao Ding

**Affiliations:** 1 College of Economics, Jinan University, Guangzhou, China; 2 The Second Clinical Medical College, Guangzhou University of Chinese Medicine, Guangzhou, China; 3 The First Affiliate Hospital of Guangzhou Medical University, Guangzhou, China; 4 AI Lab, Yidu Cloud (Beijing) Technology Co. Ltd., Beijing, China; 5 School of Computer Science, South China Normal University, Guangzhou, China; 6 School of Information Science and Technology, Guangdong University of Foreign Studies, Guangzhou, China

**Keywords:** Artificial intelligence, Electronic health records, Bibliometrics, Topic modelling, United States, China

## Abstract

**Background:**

The application of artificial intelligence techniques for processing electronic health records data plays increasingly significant role in advancing clinical decision support. This study conducts a quantitative comparison on the research of utilizing artificial intelligence on electronic health records between the USA and China to discovery their research similarities and differences.

**Methods:**

Publications from both Web of Science and PubMed are retrieved to explore the research status and academic performances of the two countries quantitatively. Bibliometrics, geographic visualization, collaboration degree calculation, social network analysis, latent dirichlet allocation, and affinity propagation clustering are applied to analyze research quantity, collaboration relations, and hot research topics.

**Results:**

There are 1031 publications from the USA and 173 publications from China during 2008–2017 period. The annual numbers of publications from the USA and China increase polynomially. *JAMIA* with 135 publications and *JBI* with 13 publications are the top prolific journals for the USA and China, respectively. *Harvard University* with 101 publications and *Zhejiang University* with 12 publications are the top prolific affiliations for the USA and China, respectively. *Massachusetts* is the most prolific region with 211 publications for the USA, while for China, *Taiwan* is the top 1 with 47 publications. China has relatively higher institutional and international collaborations. Nine main research areas for the USA are identified, differentiating 7 for China.

**Conclusions:**

There is a steadily growing presence and increasing visibility of utilizing artificial intelligence on electronic health records for the USA and China over the years. The results of the study demonstrate the research similarities and differences, as well as strengths and weaknesses of the two countries.

## Background

With the expanding use and increasing possibility of including information relating to patient outcomes and functionality such as clinical decision support, Electronic Health Records (EHRs) becomes increasingly valuable information about patient health conditions and responses to treatment over time [1]. The field of utilizing artificial intelligence techniques on EHRs data processing has attracted increasing interests from scientific community, reflected by the increasing of publications from major scientific literature databases such as Web of Science (WoS) and PubMed. The USA and China are top 2 largest economies in the world. According to literature retrieval in WoS, the two countries have the most publications the field in the last decade. Therefore, it is meaningful to conduct a quantitative analysis of the research publications from the two countries to compare their research similarities and differences, as well as strengths and weaknesses.

Research publication plays an important role in providing key linkage between knowledge generation, uptake and use in the scientific process [2]. Bibliometrics involves statistical analysis of written publications. It has been the method of choice for quantitative assessments of academic research to comprehensively explore the research advances in the past and identify future research trends in a specific field [3]. Bibliographic data from citation indexes, e.g., titles, journal, abstracts, author addresses, and etc., are analyzed statistically to recognize the popularity and impact of specific publications, authors, affiliations, or an entire field. Bibliometrics has been widely performed in the evaluation of various research areas [4, 5]. Especially, it has also been adopted to the evolution of interdisciplinary research field, e.g., natural language processing in medical research [6], natural language processing empowered mobile computing research [7], technology enhanced language learning research [8], and text mining in medical research [9].

To that end, relevant publications in the field were retrieved from both WoS and PubMed to quantitatively explore the academic performances of the two countries in terms of current research status, research intellectual structures, and research focuses. Analyzing techniques include bibliometrics, geographic visualization, collaboration degree calculation, social network analysis, latent dirichlet allocation, and affinity propagation clustering.

Specifically, the following comparisons are conducted: 1) studying the quantitative distributions and growth characteristics of the publications, 2) identifying prolific publication sources, authors, and affiliations, 3) exploring publication geographical distributions, 4) investigating collaboration degrees and collaboration patterns, 5) visualizing scientific collaboration relations, and 6) discovering hot research topics and topic evolutions.

## Methods

### Data sources

The publications in the research field during 2008–2017 from WoS and PubMed databases were preferred. With a list of search keywords determined by domain experts, as shown in Table 1, publications with “Article” type were retrieved and downloaded as plain texts. After manual review, 1031 records from the USA and 173 records from China were obtained for comparison analysis. Key elements including title, publication year, keywords, abstract, author address were extracted. In addition, corresponding affiliations and regions were automatically extracted from author address information. Key words from author keywords, Keywords Plus/PubMed MeSH, title, and abstract, were extracted by our developed natural language processing module.

**Table 1:** Search keywords related to “artificial intelligence” and “EHR”

Keywords related to “artificial intelligence”	“artificial intelligence” OR “intelligent information processing” OR “machine learning” OR “pattern recognition” OR “information retrieval” OR “information extraction” OR “data mining” OR “text mining” OR “deep learning” OR “neural network” OR “natural language processing” OR “NLP” OR “semantic analysis” OR “question answering” OR “word sense disambiguation” OR “named entity recognition” OR “language modeling” OR “intelligent computing” OR “intelligent computation” OR “speech recognition” OR “smart learning” OR “knowledge graph” OR “automated reasoning” OR “automated inference” OR “knowledge representation” OR “fuzzy logic” OR “bayesian network” OR “machine intelligence” OR “natural language generation” OR “natural language understanding” OR “bayesian networks” OR “neural networks” OR “classification algorithm” OR “clustering algorithm” OR “association rule mining”
Keywords related to “EHRs”	“electronic medical record” OR “clinical notes” OR “clinical summary” OR “discharge summary” OR “EMR” OR “medical data” OR “electronic patient record” OR “medical record” OR “medical records” OR “electronic medical records” OR “electronic health record” OR “EHR” OR “electronic health records” OR “EHRs” OR “EMRs” OR “clinical note” OR “electronic patient records” OR “personal health record”

In addition to basic bibliometric analysis, the techniques used in this paper include: geographic visualization, co-authorship index and collaboration degree calculation, social network analysis, and topic modelling analysis.

### Geographic visualization analysis

Geographic visualization [10] refers to a set of visualization technologies for supporting geospatial data analysis. It provides ways to explore both the information display and the data behind the information itself to more readily view complex relations in images [11, 12]. Geographic visualization works essentially by helping people see the unseen more effectively in a visual environment than when using textual or numerical description. In this study, we apply geographic visualization analysis to explore publication geographical distributions in the USA and China, respectively.

### Co-authorship index and collaboration degree

Co-authorship index shown as Eq. (1), was firstly elaborated by Schubert and Braun [13]. It is obtained by calculating proportionally the publications co-authored by single, two, multi- and mega-authors for different countries. Here, the publications have been firstly divided into four categories according to author count, i.e., single-author, two-author, multiple-author publications with three to four authors, and mega-author publications with five or more authors.

1 $$ CAI=\frac{\left({N}_{\mathrm{ij}}/{N}_{io}\right)}{\left({N}_{oj}/{N}_{oo}\right)}\times 100 $$

In the equation, *N*_*ij*_ is the publication count co-authored by *j* authors in the *i*^*th*^ country, *N*_*io*_ is the publication count in the *i*^*th*^ country, *N*_*oj*_ is the publication count co-authored by *j* authors in all countries, *N*_*oo*_ is the publication count in all countries. *CAI* = 100 represents the average level. *CAI* > 100 indicates higher than the average, while *CAI* < 100 reflects lower than the average.

As a measure of scientific research’s connective relation to the level of author, affiliation, or country, the collaboration degree can be calculated as Eq. (2) [14, 15].

2 $$ {C}_{Ai}=\frac{\sum_{j=1}^N{\alpha}_j}{N} $$

In the equation, *C*_*Ai*_ indicates the collaboration degree of the *i* year in the author, affiliation or country level. *α*_*j*_ donates the count of author, affiliation or country for each publication. *N* is the annual publication count.

In this study, co-authorship index is used to study collaboration patterns of authors, and collaboration degree is applied to measure the scientific research’s connective relation to the three levels.

### Social network analysis

Social network analysis (SNA) focuses on the structure of ties within, e.g., persons, organizations, or the products of human activity or cognition such as web sites [16]. SNA works based mainly on networks and graph theory [17], and it provides both a visual and a mathematical analysis of human relations. In this study, the collaboration relations for authors, affiliations and countries are explored using social network analysis. In the network, the nodes are specific authors, affiliations or countries, and the lines are the collaboration relations. The size of node indicates the publication count of a specific author, affiliation or country. The width of link indicates the collaboration frequency between the two authors, affiliations or countries.

### Topic modelling analysis

Topic modeling extracts semantic information from a collection of texts using statistical algorithms. Latent Dirichlet Allocation (LDA) is an improved three-layer Bayesian model developed by Blei et al. [18]. In LDA, each document in the text corpus is modeled as a set of draws from a mixture distribution over a set of hidden topics, where topics are assumed to be uncorrelated and each is characterized by a distribution over words. In LDA, a *word* is defined as an item from a vocabulary indexed by {1, …, *V*}, a *document* is a sequence of *N* words denoted by *d* = (*w*_1_, …, *w*_*N*_), and a *corpus* is a collection of *M* documents denoted by *D* = {*d*_1_, …, *d*_*M*_}. The generation process is as follows: 1) The term distribution *β* indicating the probability of a word occurring in a given topic is as *β*~*Dirichlet*(*δ*); 2) The proportions *θ* of the topic distribution for a document *d* are determined by *θ*~*Dirichlet*(*α*); 3) A topic is chosen by the distribution *z*_*i*_~*Multinomial*(*θ*) for each word *w*_*i*_ in the document *d*, and a word is chosen from a multinomial probability distribution conditioned on the topic *z*_*i*_ : *p*(*w*_*i*_| *z*_*i*_, *β*). As for variational expectation-maximization, the log-likelihood for one document *d* ∈ *D* is given by Eq. (3), and the likelihood for Gibbs sampling estimation with *k* topics is as Eq. (4).

3 $$ \ell \left(\alpha, \beta \right)=\log \left(p\left(d|\alpha, \beta \right)\right)=\log \int \left\{{\sum}_z\left[{\prod}_{i=1}^Np\left({w}_i|{z}_i,\beta \right)p\left({z}_i|\theta \right)\right]\right\}p\left(\theta |\alpha \right) d\theta $$

4 $$ \log \left(p\left(d|z\right)\right)=k\log \left(\frac{\varGamma \left( V\delta \right)}{\varGamma {\left(\delta \right)}^V}\right)+{\sum}_{K=1}^k\left\{\left[{\sum}_{j=1}^V\log \left(\varGamma \left({n}_K^{(j)}+\delta \right)\right)\right.-\log \left(\varGamma \left({n}_K^{(.)}+ V\delta \right)\right)\right. $$

Further, Affinity Propagation (AP) clustering is used for the cluster analysis of the topics identified by LDA. AP was proposed by Frey and Dueck [19] with a basis of message passing. It does not require users to set cluster count in advance, but considers all data points to be potential exemplars and transmits real-valued messages recursively until a set exemplars of high-quality emerges [20]. AP was found to identify clusters with lower error rate and less time [21].

AP calculates the “responsibility” *r*(*i*, *k*) and the “availability” *a*(*i*, *k*), shown as Eqs. (5) and (6) for each node *i* and each candidate exemplar *k*. *r*(*i*, *k*) is the suitableness of *k* as an exemplar for *i*, while *a*(*i*, *k*) is the evidence that *i* should choose *k* as an exemplar.

5 $$ r\left(i,k\right)\leftarrow s\left(i,k\right)-\underset{k^{\prime }:{k}^{\prime}\ne k}{\max}\left\{a\left(i,{k}^{\prime}\right)+s\left(i,{k}^{\prime}\right)\right\} $$

6 $$ a\left(i,k\right)\leftarrow \min 0,r\left(k,k\right)+{\sum}_{i^{\prime }:{i}^{\prime}\notin \left\{i,k\right\}}\max \left\{0,r\left({i}^{\prime },k\right)\right\} $$

In the equations, *s*(*i*, *k*) is the similarity between two nodes *i* and *k*. When a good set of exemplars emerges, Eqs. (5) and (6) will stop iterating. Each node *i* can then be assigned to the exemplar *k* that maximizes *a*(*i*, *k*) + *r*(*i*, *k*). If *i* = *k*, then *i* is an exemplar. Numerical oscillations is controlled using a damping factor between 0 and 1.

In this study, words from author keywords and Keywords Plus/PubMed MeSH, publication title, as well as abstract with weights 0.4, 0.4, and 0.2 determined by our former study [6] are used as analysis units in topic modelling analysis. Term Frequency-Inverse Document Frequencies (TF-IDF) is used to filter out unimportant terms.

## Results

### Growth of publications

**Fig. 1:**
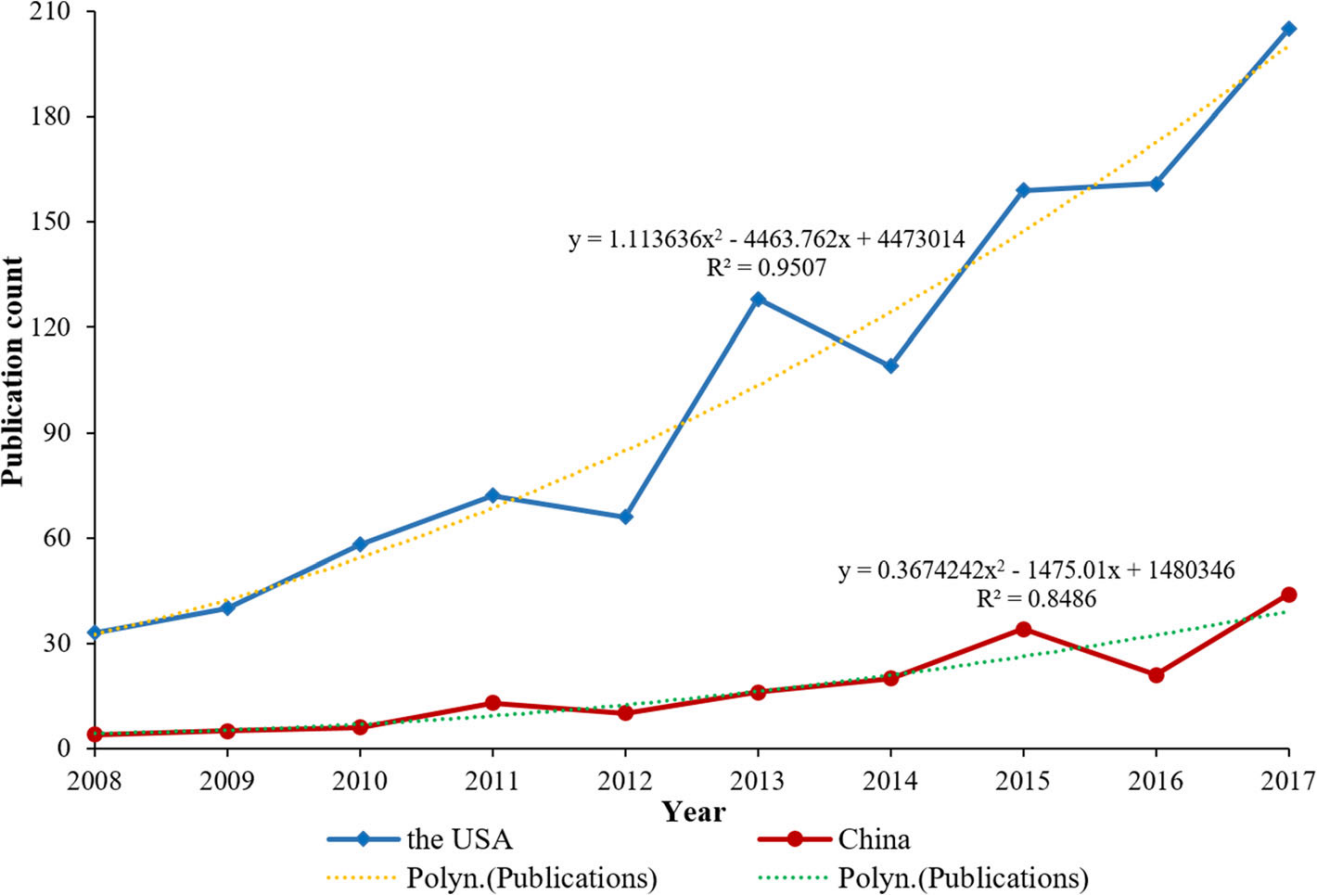
The distributions of total publications by year

The distributions of total publications by year for the USA and China are shown in Fig. 1**.** The publication counts for both two countries are overall showing increasing trends in fluctuation. The average publications during the study period are 103.1 and 17.3 articles per year. The highest productivity is observed in 2017 with a total of 205 (19.88%) articles for the USA and 44 (25.43%) articles for China. The annual growth rates reach 26.18 and 40.54% on average for the USA and China, respectively. The trend of publications for the USA is similar with the polynomial curve (*p* < 0.05, *R*^2^ = 95.07%) expressed as *y* = 1.113636*x*2 − 4463.762*x* + 4473014, while the publication trend for China is similar with the polynomial curve (*p* < 0.05, *R*^2^ = 84.86%) expressed as *z* = 0.3674242*x*2 − 1475.01*x* + 1480346. With the simulation curves, the future productivity can be predicted. The predictive values for year 2018 for the USA and China are 230 and 47, respectively.

### Prolific publication sources

The 1031 records from the USA are published in 347 unique journal or conference proceeding sources, and 92 publication sources contribute to China’s 173 publications. The top 16 publication sources for the USA in Table 2 account for 49.08% of the total publications, and the 14 prolific ones for China contribute to 43.35% of the total publications. The top 3 publication sources for the USA are *Journal of the American Medical Informatics Association*, *Journal of Biomedical Informatics*, and *AMIA Annual Symposium Proceedings*. As for China, the top 3 prolific ones are *Journal of Biomedical Informatics*, *Journal of Biomedical Engineering*, and *Studies in Health Technology and Informatics*.

**Table 2:** Prolific publication sources

R	Prolific publication sources for the USA	TP	P%	R	Prolific publication sources for China	TP	P%
1	*Journal of the American Medical Informatics Association*	135	13.09	1	*Journal of Biomedical Informatics*	13	7.51
2	*Journal of Biomedical Informatics*	96	9.31	2	*Journal of Biomedical Engineering*	6	3.47
3	*AMIA Annual Symposium Proceedings*	65	6.30	3	*Studies in Health Technology and Informatics*	6	3.47
4	*Studies in Health Technology and Informatics*	29	2.81	4	*China Journal of Chinese Materia Medica*	6	3.47
5	*International Journal of Medical Informatics*	28	2.72	5	*Computer Methods and Programs in Biomedicine*	5	2.89
6	*PLoS One*	27	2.62	6	*Expert Systems with Applications*	5	2.89
7	*BMC Medical Informatics and Decision Making*	20	1.94	7	*IEEE Access*	5	2.89
8	*AMIA Joint Summits on Translational Science Proceedings*	19	1.84	8	*Journal of the American Medical Informatics Association*	5	2.89
9	*Applied Clinical Informatics*	18	1.75	9	*Artificial Intelligence in Medicine*	4	2.31
10	*Artificial Intelligence in Medicine*	14	1.36	10	*BMC Medical Informatics and Decision Making*	4	2.31
11	*JMIR Medical Informatics*	12	1.16	11	*Journal of Medical Systems*	4	2.31
12	*Journal of Biomedical Semantics*	10	0.97	12	*Knowledge-based Systems*	4	2.31
13	*Yearbook of Medical Informatics*	9	0.87	13	*PLoS One*	4	2.31
14	*IEEE Journal of Biomedical and Health Informatics*	8	0.78	14	*Chinese Journal of Integrated Traditional and Western Medicine*	4	2.31
15	*Journal of Medical Systems*	8	0.78				
16	*Medical Care*	8	0.78				

### Prolific authors and affiliations

Three thousand three hundred fifty authors and 542 affiliations from the USA contribute to the 1031 publications, and 635 authors and 208 affiliations from China for the 173 publications. Table 3 shows prolific authors with *Joshua C. Denny* (53 publications), *Hongfang Liu* (36 publications), *Guergana Savova* (34 publications), *Hua Xu* (32 publications), and *Christopher G. Chute* (28 publications) as the top 5 for the USA. As for China, *Buzhou Tang* (7 publications) and *Jianbo Lei* (6 publications) are the top 2. Table 4 lists top prolific affiliations, where *Harvard University* with 101 publications is ranked at 1st for the USA. Other prolific affiliations include *Vanderbilt University* with 96 publications and *Mayo Clinic* with 93 publications. As for China, the top 3 are *Zhejiang University*, *National Taiwan University*, and *China Academy of Chinese Medical Sciences*.

**Table 3:** Top prolific authors

the USA				China			
Rank	Name	Country	TP	Rank	Name	Country	TP
1	*Joshua C. Denny*	the USA	53	1	*Buzhou Tang*	China	7
2	*Hongfang Liu*	the USA	36	2	*Jianbo Lei*	China	6
3	*Guergana Savova*	the USA	34	3	*Hong-Jie Dai*	China	4
4	*Hua Xu*	the USA	32	4	*Huabing Zhang*	China	4
5	*Christopher G. Chute*	the USA	28	5	*Jingchi Jiang*	China	4
6	*Nigam H. Shah*	the USA	22	6	*Qingcai Chen*	China	4
7	*Matthew Samore*	the USA	21	7	*Simon Fong*	China	4
8	*Isaac S. Kohane*	the USA	20	8	*Xiaolong Wang*	China	4
9	*Shawn N. Murphy*	the USA	19	9	*Yi Guan*	China	4
10	*Carol Friedman*	the USA	18	10	*Zengjian Liu*	China	4
11	*Peter Szolovits*	the USA	18	11	*Zhengxing Huang*	China	4

**Table 4:** Top prolific affiliations

Rank	Name	Country	TP	Rank	Name	Country	TP
1	*Harvard University*	the USA	101	1	*Zhejiang University*	China	12
2	*Vanderbilt University*	the USA	96	2	*National Taiwan University*	China	10
3	*Mayo Clinic*	the USA	93	3	*China Academy of Chinese Medical Sciences*	China	9
4	*University of Utah*	the USA	82	4	*Peking University*	China	8
5	*Columbia University*	the USA	72	5	*Tsinghua University*	China	8
6	*Brigham and Women’s Hospital*	the USA	63	6	*Chinese Academy of Sciences*	China	7
7	*Stanford University*	the USA	53	7	*Harbin Institute of Technology, Shenzhen*	China	7
8	*Massachusetts General Hospital*	the USA	48	8	*National Taiwan University Hospital*	China	6
9	*Partners Healthcare Inc*	the USA	48	9	*Shanghai Jiao Tong University*	China	6
10	*University of Texas at Houston*	the USA	43	10	*University of Macau*	China	6

### Geographical distribution of publications

**Fig. 2:**
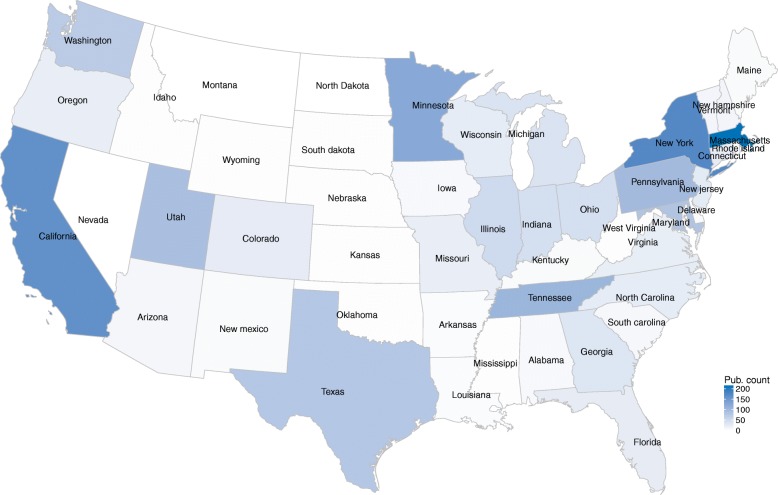
Geographical distributions of the publications in the USA

**Fig. 3:**
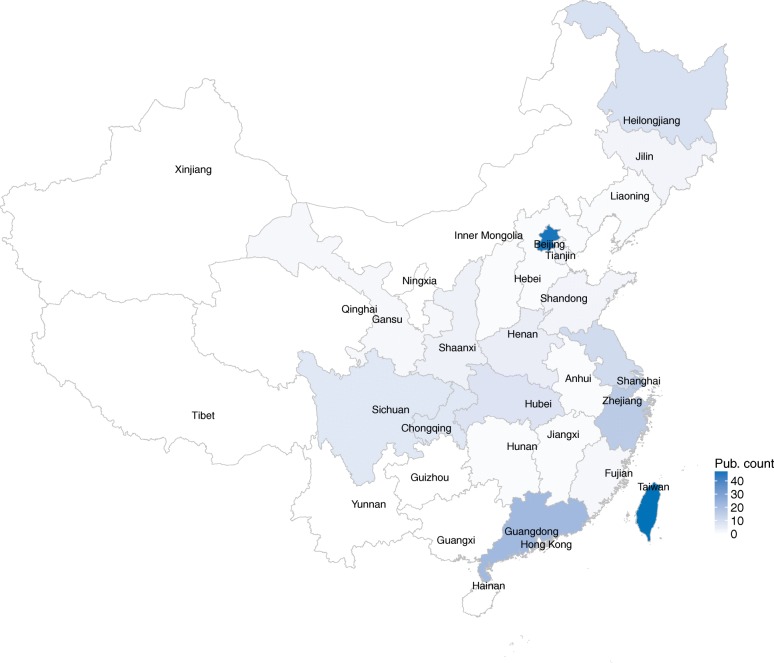
Geographical distributions of the publications in China

We study the concentration of researches in the USA and China at regional levels. The spatial characteristics of the publications from the two countries are explored. 46 states in the USA involve in the 1031 publications and 25 regions in China contribute to the 173 publications. The geographical distributions are shown as Figs. 2 and 3, respectively. The figures display that the USA and China’ publications vary widely across the whole country. As for the USA, the top 5 prolific states are Massachusetts (211 publications), New York (173 publications), California (161 publications), Minnesota (122 publications), and Tennessee (102 publications). As for China, the top 5 regions are Taiwan (47 publications), Beijing (46 publications), Guangdong (22 publications), Shanghai (17 publications), and Zhejiang (16 publications). The publications authored by Chinese and the USA’s scholars are shown in Table 5 by top regions. For exploring the structures and dynamics of the publications, we split the whole period into two 5-year phases: 2008–2012 and 2013–2017. In the two different phases, Massachusetts, New York, California, and Minnesota always appear among the top 5 for the USA. As for China, Taiwan and Beijing are always at the top 2 places.

**Table 5:** Regional distributions of publications

Country	the USA						China					
Period Num.	2008–2017 1031		2008–2012 269		2013–2017 762		2008–2017 173		2008–2012 38		2013–2017 135	
Rank	Region	Num.	Region	Num.	Region	Num.	Region	Num.	Region	Num.	Region	Num.
1	Massachusetts	211	New York	45	Massachusetts	169	Taiwan	47	Taiwan	15	Beijing	38
2	New York	173	Massachusetts	42	California	129	Beijing	46	Beijing	8	Taiwan	32
3	California	161	Minnesota	37	New York	128	Guangdong	22	Hong Kong	3	Guangdong	21
4	Minnesota	122	Tennessee	36	Minnesota	85	Shanghai	17	Sichuan	3	Shanghai	16
5	Tennessee	102	California	32	Pennsylvania	81	Zhejiang	16	Zhejiang	3	Zhejiang	13
6	Pennsylvania	98	Utah	27	Texas	68	Jiangsu	11	Heilongjiang	2	Jiangsu	10
7	Utah	90	Maryland	17	Tennessee	66	Heilongjiang	9	Macau	2	Hubei	8
8	Maryland	81	Pennsylvania	17	Maryland	64	Hubei	8	Chongqing	1	Heilongjiang	7
9	Texas	78	Washington	16	Utah	63	Chongqing	7	Gansu	1	Chongqing	6
10	Washington	72	Indiana	14	Washington	56	Sichuan	7	Guangdong	1	Henan	5
11	Illinois	51	Wisconsin	13	Ohio	42	Hong Kong	6	Jiangsu	1	Macau	4
12	Indiana	45	Illinois	11	Illinois	40	Macau	6	Shaanxi	1	Sichuan	4
13	Ohio	45	Florida	10	Indiana	31	Henan	5	Shandong	1	Hong Kong	3
14	Michigan	38	Michigan	10	Michigan	28	Shaanxi	4	Shanghai	1	Jilin	3

### Authorship pattern and collaboration

**Fig. 4:**
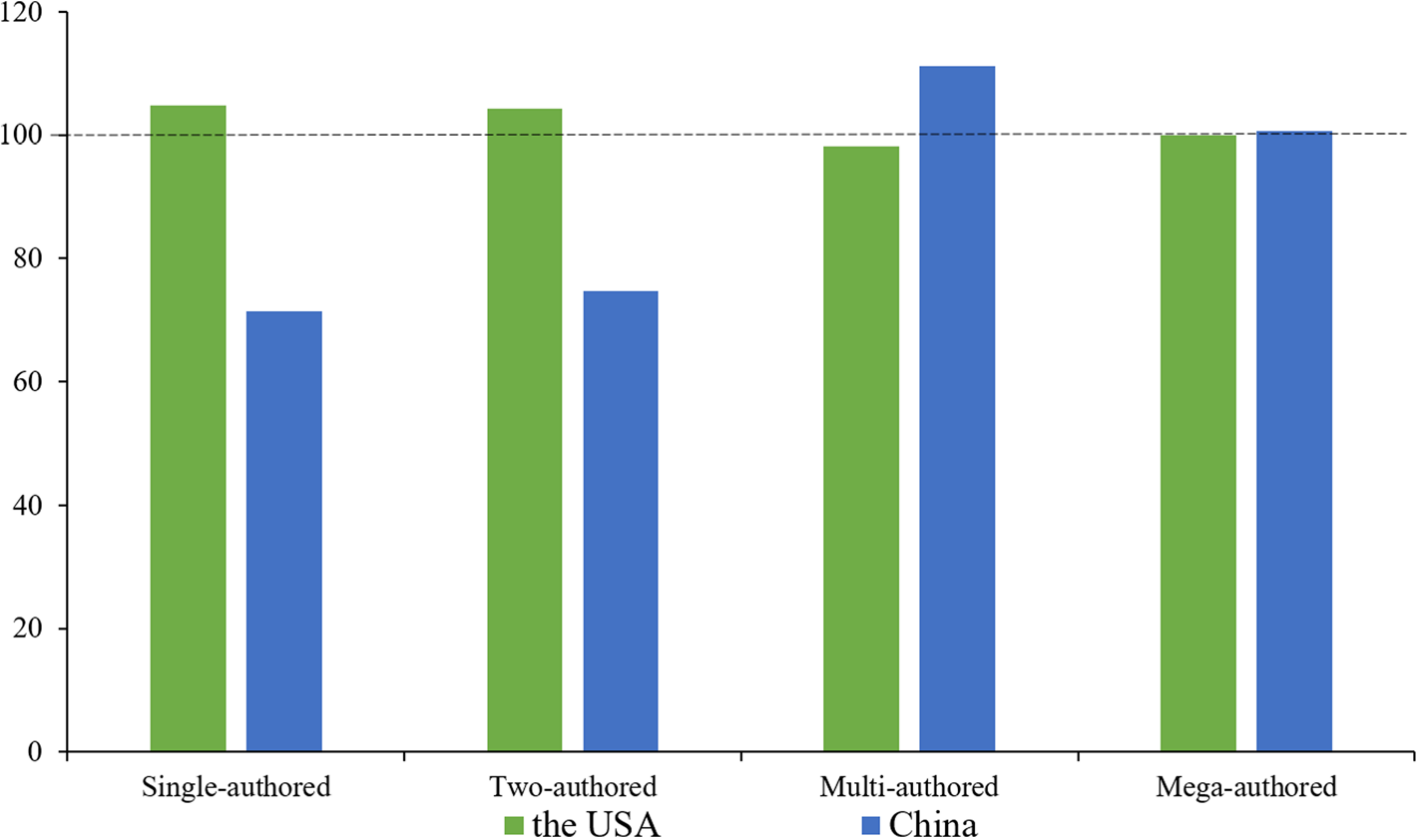
Sketch map of collaboration patterns reflected by *CAI*

**Fig. 5:**
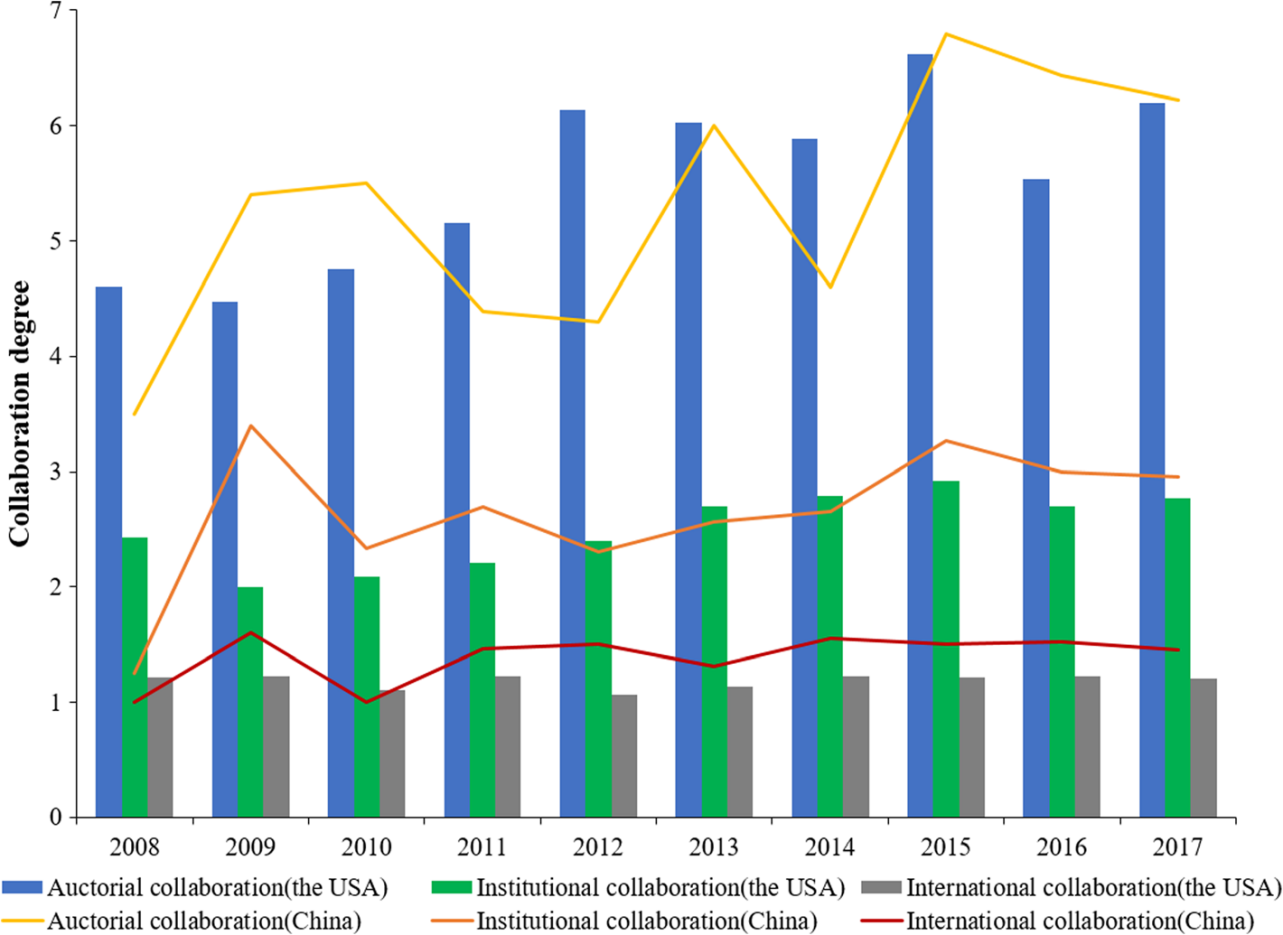
Annual collaboration degree distributions

The profiles of *CAI* for the USA and China have been illustrated in Fig. 4. It is clearly indicated that *CAIs* of multi- and mega-author publications in the research filed in China are slightly higher than the average. However, the *CAIs* of multi- and mega-author publications in the USA are lower than the average. Figure 5 shows the collaboration degrees at the country, affiliation and author levels in the two countries. On the whole, the international collaboration degree is growing relatively slowly than the author and affiliation collaboration degrees. On average, 5.83 authors, 2.63 affiliations and 1.18 countries participate in each publication from the USA. As for China, on average each publication has 5.79 authors, 2.84 affiliations and 1.39 countries. The average degrees of affiliation and country for China’s publications are higher than that for the USA’s publications, while the average degrees of author is on the contrary.

**Fig. 6:**
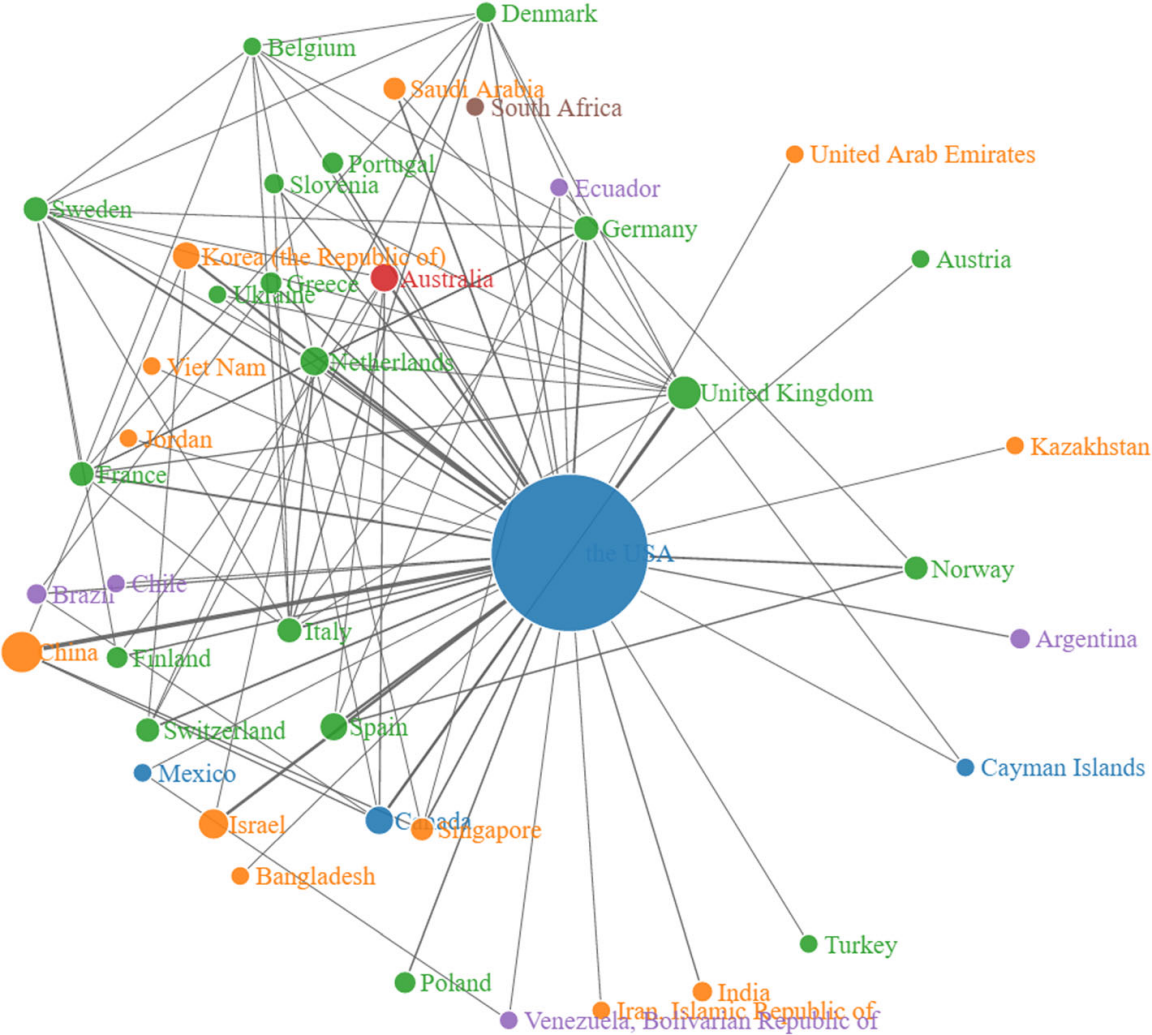
Collaboration network in country level for the USA’s publications

The collaboration among countries/regions for the USA’s publications is then visualized as Fig. 6 (access via the link [22]). From the figure, the USA (the largest node in blue color) in the center of the network has the most collaborations with other countries/regions. The USA-China collaboration (the thickest line) is ranked at 1st. The collaboration networks among affiliations with publications > = 15 (access via the link [23]) and among authors with publications > = 12 (access via the link [24]) are also visualized. Furthermore, we also visualize the collaborations for China’s publications including country/region collaboration (access via the link [25]), collaboration among affiliations with publications > = 3 (access via the link [26]), and collaboration among authors with publications > = 3 (access via the link [27]). By accessing to the dynamic networks, through simply clicking the nodes, users can explore the collaboration relations for specific countries/regions, affiliations, or authors.

### Topic generation and clustering

**Fig. 7:**
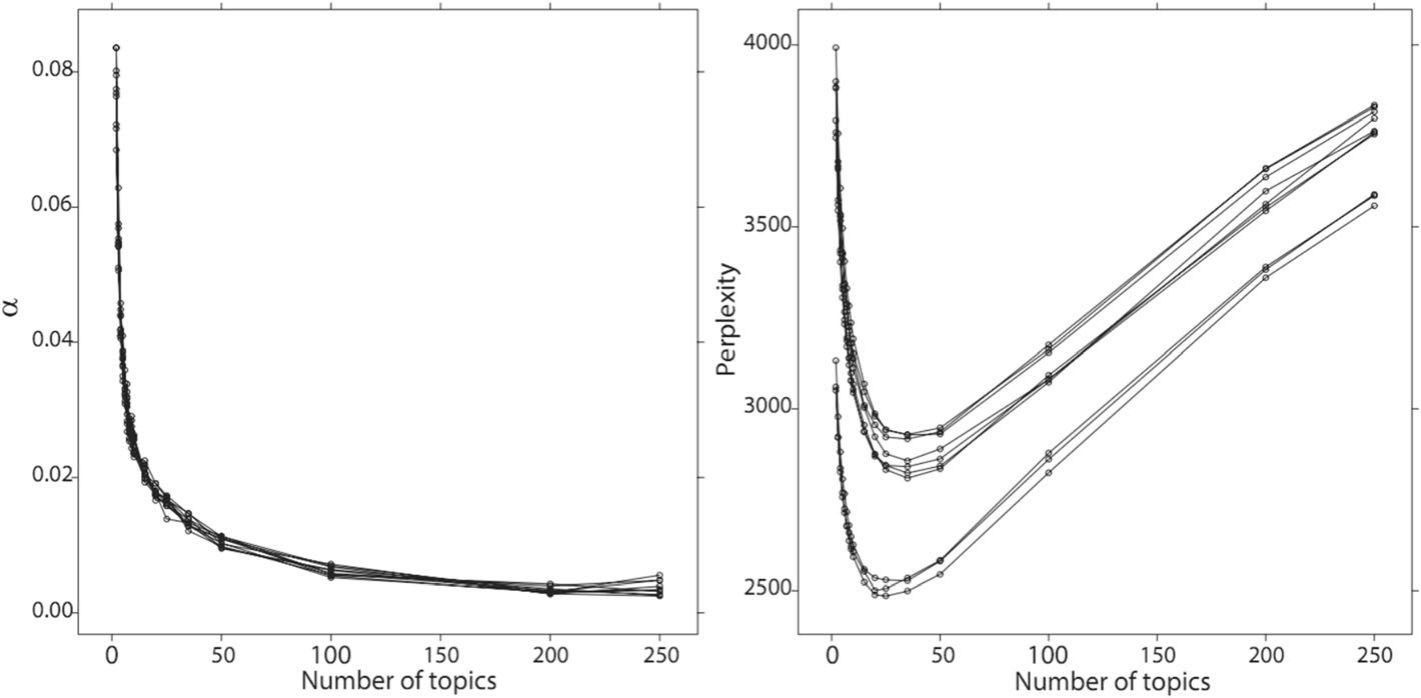
Left: estimated *α* value for the models fitted using VEM. Right: perplexities of the test data for the models fitted by using Gibbs sampling. Each line corresponded to one of the folds in the 10-fold cross-validation for the USA’s publications

**Fig. 8:**
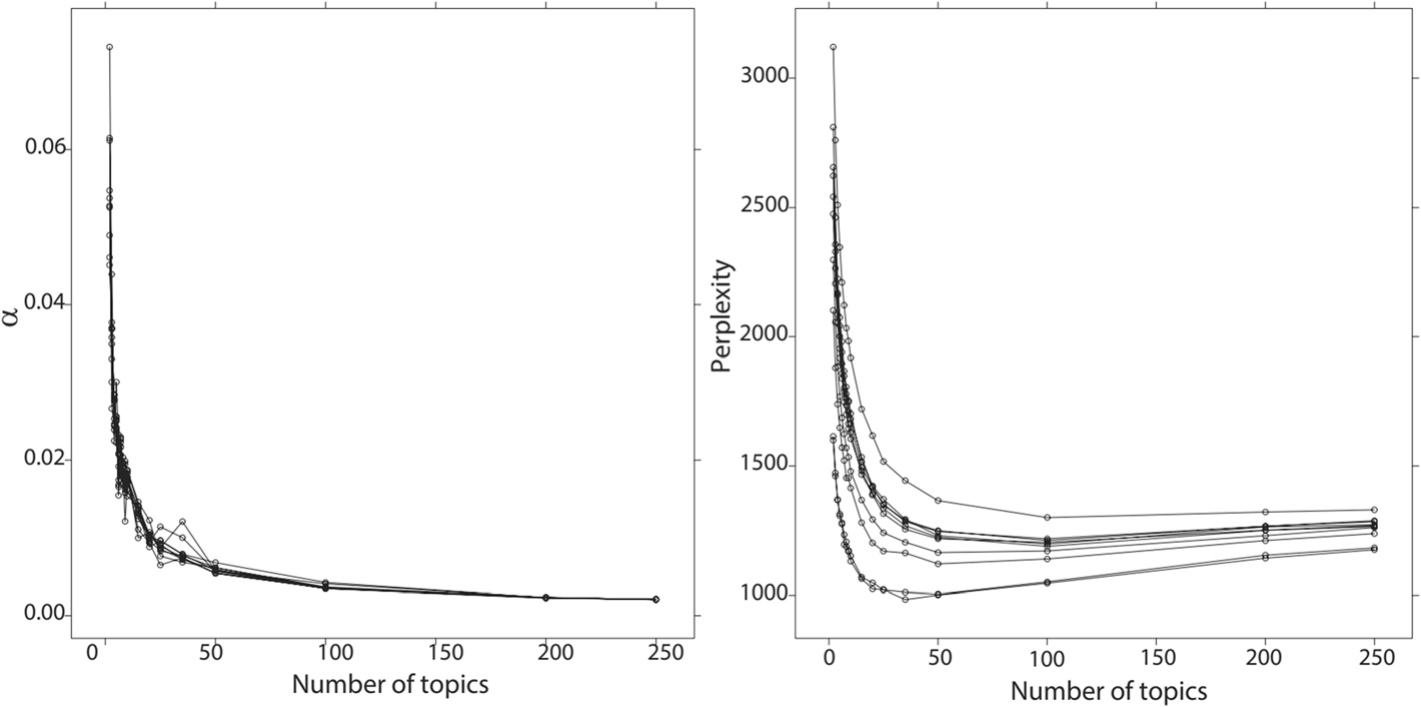
Left: estimated *α* value for the models fitted using VEM. Right: perplexities of the test data for the models fitted by using Gibbs sampling. Each line corresponded to one of the folds in the 10-fold cross-validation for China’s publications

**Fig. 9:**
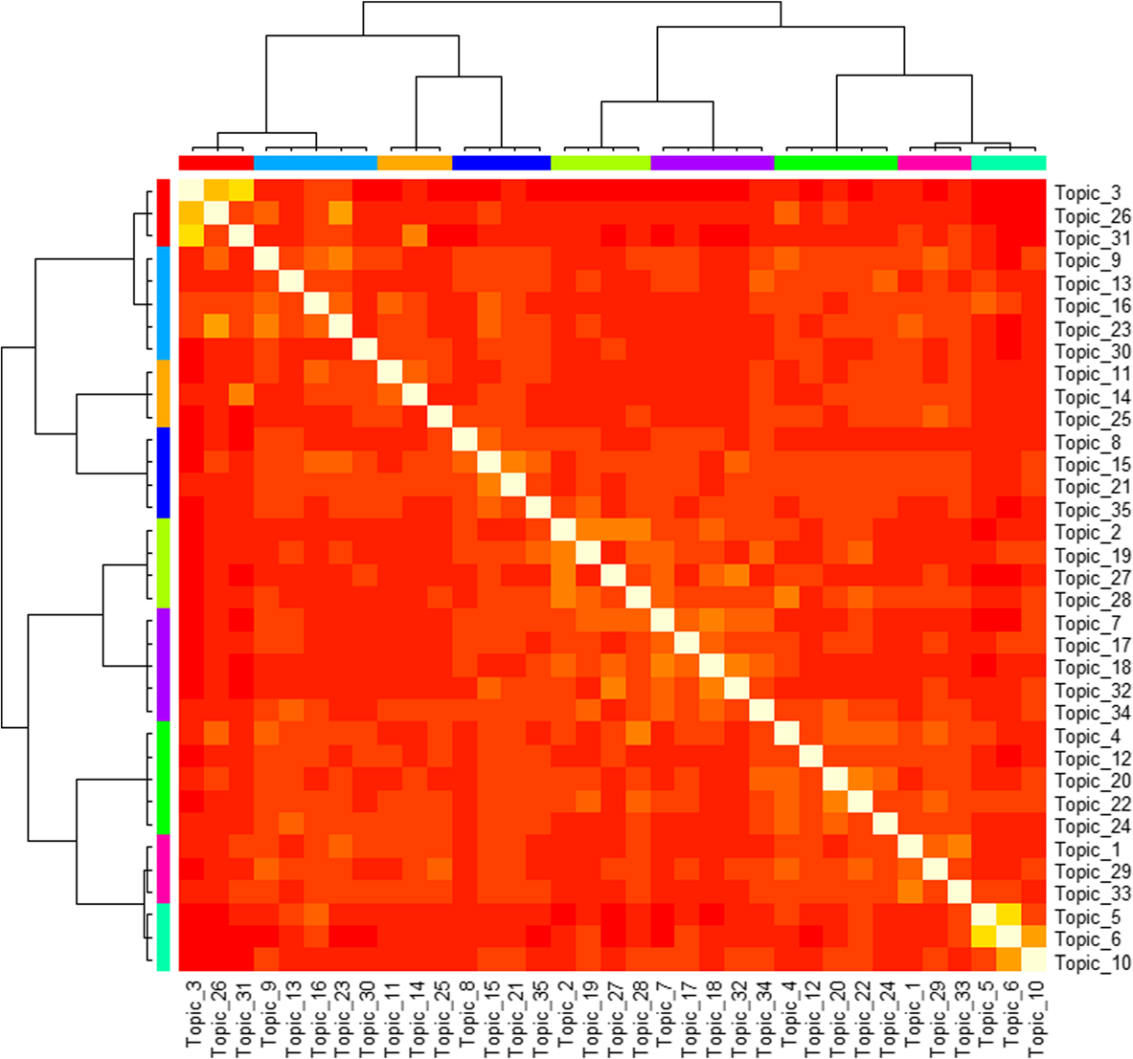
AP clustering result of the identified clusters for the USA’s publications

**Fig. 10:**
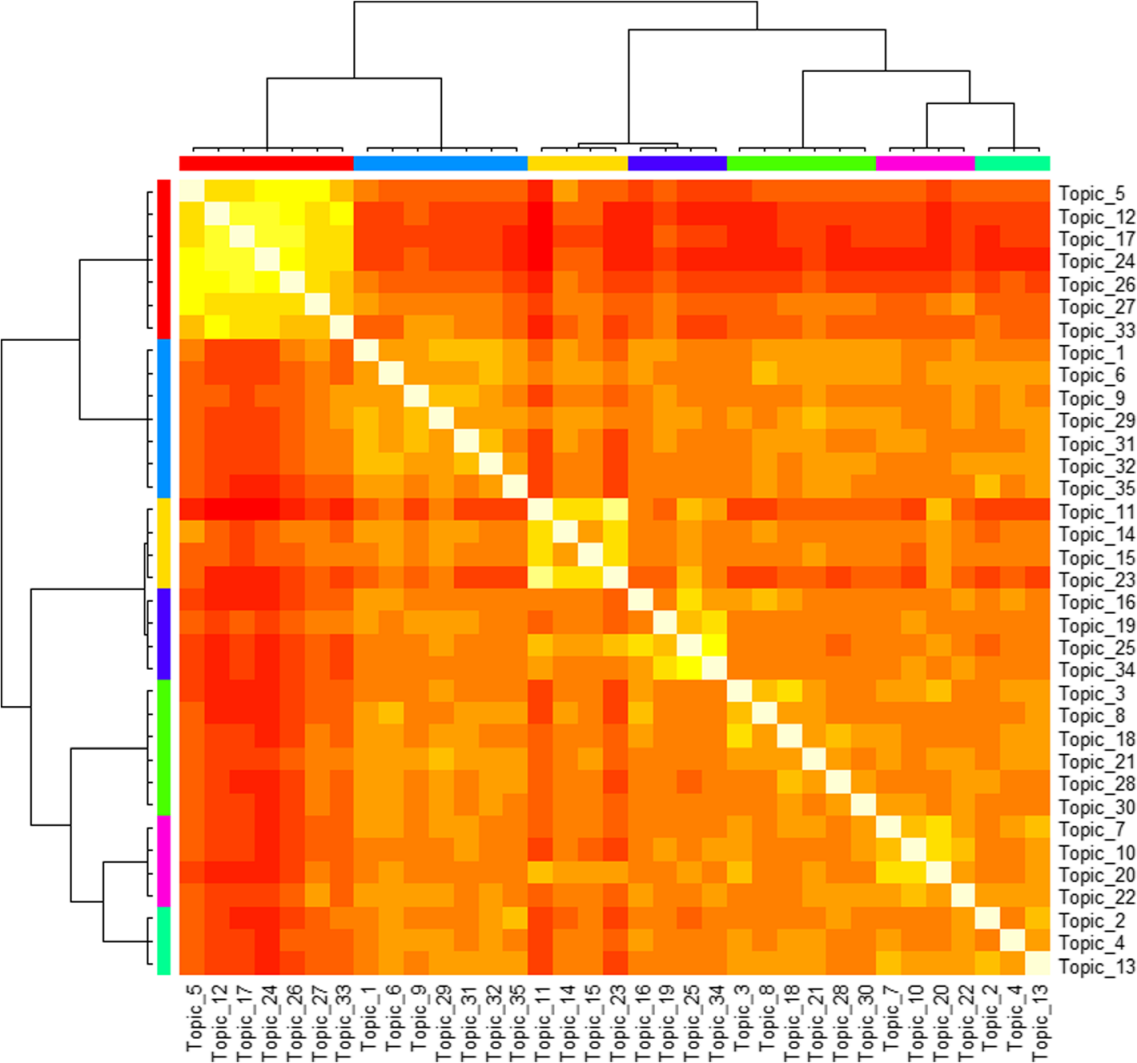
AP clustering result of the identified clusters for China’s publications

**Fig. 11:**
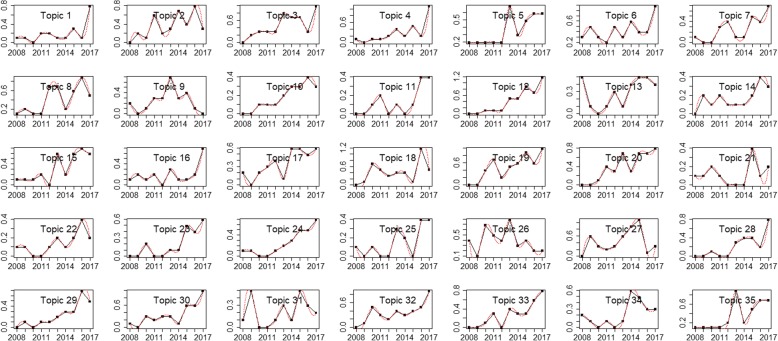
The trends of research topics for the USA’s publications

**Fig. 12:**
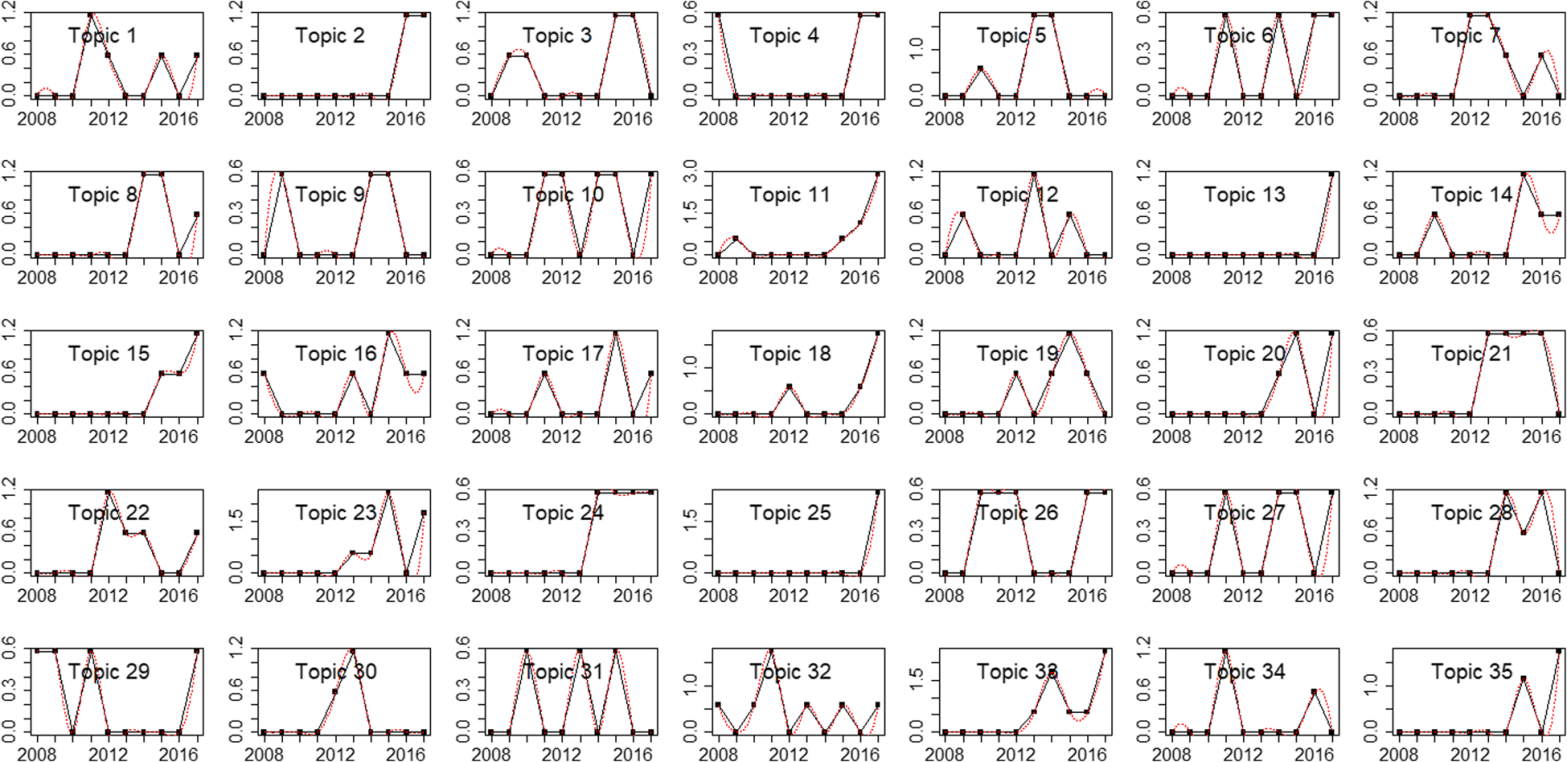
The trends of research topics for China’s publications

By setting TF-IDF value threshold as 0.1, top used terms in the author keywords, Keywords Plus/PubMed MeSH, title, and abstract of the publications are ranked by frequency. The top 5 terms and their frequencies for the USA are *Drug* (483), *Medication* (411), *Cancer* (370), *Adverse* (362), and *Phenotype* (275), while the top terms for China are *Risk* (195), *Medicine* (125), *Drug* (107), *Cancer* (76), and *Diabetes* (71). Figures 7 and 8 present the perplexities of models fitted using Gibbs sampling with different topic counts. The results suggest that the optimal topic count can be set to 35 for both the USA and China. The *α* is then set to 0.01339416 for the USA and 0.008163102 for China. We estimate the LDA models using Gibbs sampling with the parameters. Potential themes are assigned to each topic through semantics analysis of representative terms and text intention reviewing. Table 6 displays the top 5 best matching topics for the USA including *Drug adverse event*, *Vaccine*, *Diabetes mellitus*, *Health data confidentiality*, and *Health data analysis technique*, while the top 5 for China are *Named entity recognition*, *Drug adverse event*, *Smoking*, *Prescription & drug*, and *Risk event*. The AP clustering results based on term-topic posterior probability matrix are shown in Figs. 9 and 10, where the 35 topics for the USA are categorized into 9 groups, and the 35 topics for China are categorized into 7 groups. For identifying emerging research topics, we firstly assign each publication to the topic with the highest posterior probability. We then explore the trends of research topics shown in Figs. 11 and 12. We also conduct Mann–Kendall test [28] to examine whether topics present increasing or decreasing trends.

**Table 6:** 15 selected top terms for the top 5 best matching topics

Country	Topic	Potential theme	Top high frequency terms
the USA	3	Drug adverse event	Drug; Adverse; Reaction; Pharmacovigilance; Safety; Signal; Adverse drug event; Interaction; allergy; Surveillance; Drug-drug; Spontaneous; Adverse drug reaction; Food and drug administration; Drug-drug interaction
	31	Vaccine	Vaccine; Safety; Adverse; Surveillance; Influenza; Vaccine adverse event reporting system; Adverse Even; Syndromic; Immunization; Emergency; Inactivated; Drug; Post-licensure; Anaphylaxis; Injection
	30	Diabetes mellitus	Diabetes; Mellitus; Diabetic; Ensemble; Visualization; Deterioration; Fit; Neural; Type 2 diabetes mellitus; Insulin; Support vector machine; Warning; Metformin; Glucose; Nephropathy
	27	Health data confidentiality	De-identification; Annotation; Corpus; Protected health information; Privacy; Annotator; Confidentiality; Comorbidity; Portability; Obesity; Security; Track; Anonymization; Veterans health administration; Health insurance portability and accountability act
	18	Health data analysis technique	Semantic; Terminology; Ontology; Similarity; Biomedical; Unified medical language system; Corpus; Mapping; Topic; Redundancy; Lexicon; Reasoning; Relatedness; Lexical; Nomenclature
China	33	Named entity recognition	Chinese; Entity; Word; Note; Discharge; Embedding; Annotation; Segmentation; Negation; Speculation; Conditional; Named entity recognition; Character; Deep; F-measure
	23	Drug adverse event	Risk; Statin; Adverse; Discontinuation; Cardiovascular; Event; Reaction; Heart; Coronary; Drug; Lipid-lowering; Medication; Therapy; Artery; Cardiovascular disease
	30	Smoking	Smoking; Mental; Status; Prevalence; Electric; Aged; Disorder; Open-text; Hybrid electric vehicle; CRIS-IE-Smoking; Electronic health record; Fuzzy; Logic; Bipolar; Male
	26	Prescription & drug	Prescription; Symptom; Medicine; Aspirin; Chinese; Knowledge base; Medication; Drug; Protective; Similarity; Diarrhoea; Gastrointestinal; Low-dose; Mucoprotective drug; Regularity
	14	Risk event	Congestive heart failure; Drug; Risk; Web-based; Health information exchange; Chronic; Emergency department; Deficiency; Cluster; Failure; Gastritis; Real-time; Congestive; Heart; Children of severe hand, foot, and mouth disease

## Discussion

In this study, a comparative quantitative analysis of literature of utilizing artificial intelligence on electronic health records in the USA and China are conducted. This study identifies 1031 publications from the USA and 173 publications from China for the comparative analysis. Significant and polynomial increases in publication counts for both two countries can be found. This reflects a growing interest in the research field. However, the publication count of China is not at par with that of the USA, this can also be reflected by Tables 3 and 4, where the top prolific authors and affiliations of the USA own relatively more publications than that of China. Most prolific publication sources are journals, while only some are conferences such as *AMIA Annual Symposium Proceedings*, indicating a wide influence of journal in the research field. From the publication distributions in region levels, it is obvious that for both the USA and China, most top prolific regions are also of economic prosperity.

From the authorship pattern analysis, it is found that publications published by scientists in the research field in China prefer to work in larger collaboration groups. This is consistent with the finding of Guan and Ma [29] that researchers have becoming more and more aware of the importance of collaboration. Comparatively, researchers in the USA prefer working with less collaboration. The collaboration degree analysis shows that authors or affiliations tend to collaborate more with those within the same country. Also, there are relatively more affiliations and countries participating in one publication on average for China than that for the USA. The USA and China are closest collaborators for each other.

Through topic modelling and clustering analysis, the 35 identified topics for the USA’s research are categorized into 9 areas including *Thrombosis*, *Health data privacy & confidentiality*, *Drug adverse event & vaccine*, *Imaging*, *Disease*, *Audio-visual function*, *Application of Bayesian*, *Clinical data analysis technique*, and *Nursing*. Meanwhile, the 35 identified topics for China’s research are classified into 7 areas including *Cancer*, *Imaging*, *Clinical decision support*, *Drug & risk event*, *Chinese medicine*, *Gestational diabetes mellitus*, and *Clinical data analysis techniques*. The results demonstrate the similarities and differences of the research between the two countries. From Figs. 11 and 12, as well as Mann–Kendall test, 20 topics for the USA including *Diabetes mellitus*, *Heart failure*, *Health data privacy & confidentiality*, and etc., present statistically significant increasing trends at the two-sided *p* = 0.05 level. The same is for 6 topics for China, including *Named entity recognition*, *Risk event*, *Chinese medicine*, *Brain imaging*, *Drug adverse event*, and *Cancer*. As an emerging focus in drug and cancer research topics, drug resistance has currently been one of the biggest obstacles in the treatment of cancers in clinical practice [30]. Some existing examples of cancer drug resistance research are as follows. Sun et al. [31] proposed a novel stochastic model connecting cellular mechanisms underlying cancer drug resistance to population-level patient survival for the examination of therapy-induced drug resistance and cancer metastasis. Sun and Hu [30] conducted a systematic review on the literature of mathematical modeling approaches and computational prediction methods for cancer drug resistance.

In this study, there are some limitations that are inherent to the database used and to search query developed by the authors. Such limitations were also encountered in the existing bibliometric studies, e.g., [32, 33]. Firstly, despite the fact that WoS is a widely applied repository for bibliometric analysis and PubMed is an important data source on life sciences and biomedical topics, there are still unindexed conference proceedings and journal articles. Secondly, we treat publications of journal and conference types equally important in the analysis rather than bestowing weights for publications of different types. Furthermore, since no search query is 100% perfect, thus false positive and false negative results are always a possibility. In addition, the ranking of authors and affiliations in the study is based on data presented by WoS and PubMed. However, it is possible that some authors or affiliations might have different name spelling or more than one names, which might lead to an inaccuracy in the productivity of these authors or affiliations. Despite all these limitations, our study is the first to conduct a quantitative analysis of the research publications of utilizing artificial intelligence on electronic health records from the USA and China to compare their research similarities and differences, as well as strengths and weaknesses. The findings of our study can potentially help relevant researchers, especially newcomers, understand and compare the research performance and recent development in the USA and China, especially, as well as optimize research topic decision to keep abreast of current research hotspots.

## Conclusions

Utilizing artificial intelligence techniques on EHRs research is an emerging and promising field. This research provides a most up-to-date quantitative analysis for exploring and comparing the research performance and development trends of the research field from the USA and China during the period 2008–2017. Results of this exploration present a comprehensive overview and an intellectual structure of the research, especially, research topics, for the two countries in the last decade.

## Abbreviations

AP: Affinity propagation
EHRs: Electronic Health Records
LDA: Latent dirichlet allocation
MeSH: Medical Subject Headings
SNA: Social network analysis
TF-IDF: Term Frequency-Inverse Document Frequencies
USA: United States
WoS: Web of Science

## References

[1] Patel VL, Shortliffe EH, Stefanelli M, Szolovits P, Berthold MR, Bellazzi R, Abu-Hanna A (2009). The coming of age of artificial intelligence in medicine. Artif Intell Med.

[2] Clements CJ, Watkins M, de Quadros C, Biellik R, Hadler J, McFarland D (2011). Researching routine immunization–do we know what we Don’t know?. Vaccine.

[3] Wiysonge CS, Uthman OA, Ndumbe PM, Hussey GD (2013). A bibliometric analysis of childhood immunization research productivity in Africa since the onset of the expanded program on immunization in 1974. BMC Med.

[4] Chen XL, Chen BY, Zhang CX, Hao TY (2017). Discovering the recent research in natural language processing field based on a statistical approach. Lect Notes Comput Sci.

[5] Chen XL, Weng H, Hao TY (2017). A data-driven approach for discovering the recent research status of diabetes in China. Lect Notes Comput Sci.

[6] Chen XL, Xie HR, Wang FL, Liu ZQ, Xu J, Hao TY (2018). A bibliometric analysis of natural language processing in medical research. BMC Med Inform Decis Mak.

[7] Chen XL, Ding RY, Xu K, Wang S, Hao TY, Zhou Y. A bibliometric review of natural language processing empowered mobile computing. Wirel Commun Mob Comput. 2018;2018:1-21.

[8] Chen XL, Hao JT, Hua SS, Hao TY. A bibliometric analysis of the research trends of technology enhanced language learning. Lect Notes Comput Sci. 2018;11284:169–79.

[9] Hao TY, Chen XL, Li GZ, Yan J. A Bibliometric analysis of text Mining in Medical Research. Soft Comput. 2018; pp. 1–18.

[10] Kraak MJ, Ormeling FJ. Cartography: visualization of spatial data. 3rd ed. New York: Guilford Publications; 2010.

[11] Cartwright W, Miller S, Pettit C (2004). Geographical visualization: past, present and future development. J Spat Sci.

[12] Dodge M, McDerby M, Turner M. Geographic visualization: concepts, tools and applications. Chichester: Wiley; 2008.

[13] Schubert A, Braun T (1986). Relative indicators and relational charts for comparative assessment of publication output and citation impact. Scientometrics.

[14] Zhang K, Wang Q, Liang QM, Chen H (2016). A bibliometric analysis of research on carbon tax from 1989 to 2014. Renew Sust Energ Rev.

[15] Wei YM, Mi ZF, Zhang H (2013). Progress of integrated assessment models for climate policy. Syst Eng Theory Pract.

[16] McGloin JM, Kirk DS. Social network analysis. In Handbook of quantitative criminology. New York: Springer; 2010. p. 209–24.

[17] Otte E, Rousseau R (2002). Social network analysis: a powerful strategy, also for the information sciences. J Inf Sci.

[18] Blei DM, Ng AY, Jordan MI (2003). Latent Dirichlet allocation. J Mach Learn Res.

[19] Frey BJ, Dueck D (2007). Clustering by passing messages between data points. Science.

[20] Frey BJ, Dueck D (2008). Response to comment on “clustering by passing messages between data points**”**. Science.

[21] El-Samak AF, Ashour W (2015). Optimization of traveling salesman problem using affinity propagation clustering and genetic algorithm. J Artif Intell Soft Comput Res.

[22] The Network of Countries/Regions for the USA. http://www.zhukun.org/haoty/resources.asp?id=JBMC2_US_country. Accessed 10 July 2018.

[23] The Network Affiliations with Publications >= 15 for the USA. http://www.zhukun.org/haoty/resources.asp?id=JBMC2_US_affiliation. Accessed 10 July 2018.

[24] The Network of Authors with Publications >= 12 for the USA. http://www.zhukun.org/haoty/resources.asp?id=JBMC2_US_author. Accessed 10 July 2018.

[25] The Network of Countries/Regions for China. http://www.zhukun.org/haoty/resources.asp?id=JBMC2_CN_country*.* Accessed 10 July 2018.

[26] The Network of Affiliations with Publications >= 3 for China. http://www.zhukun.org/haoty/resources.asp?id=JBMC2_CN_affiliation. Accessed 10 July 2018.

[27] The Network of Authors with Publications >= 3 for China. http://www.zhukun.org/haoty/resources.asp?id=JBMC2_CN_author. Accessed 10 July 2018.

[28] Mann HB (1945). Nonparametric tests against trend. Econometrica.

[29] Guan J, Ma NA (2004). Comparative study of research performance in computer science. Scientometrics.

[30] Sun X, Hu B. Mathematical Modeling and Computational prediction of Cancer drug resistance. Brief Bioinform. 2017. pp. 1–18. 10.1093/bib/bbx065 PMC6402530 28981626

[31] Sun X, Bao J, Shao Y (2016). Mathematical modeling of therapy-induced Cancer drug resistance: connecting Cancer mechanisms to population survival rates. Sci Rep.

[32] Sweileh M (2017). Global research trends of World Health Organization’s top eight emerging pathogens. Glob Health.

[33] Sweileh WM (2017). Bibliometric analysis of literature on toxic epidermal necrolysis and Stevens-Johnson syndrome: 1940–2015. Orphanet J Rare Dis.

